# Distorted mental spatial representation of multi-level buildings - Humans are biased towards equilateral shapes of height and width

**DOI:** 10.1038/s41598-019-50992-6

**Published:** 2019-10-21

**Authors:** M. Ertl, M. Klaus, T. Brandt, M. Dieterich, F. W. Mast

**Affiliations:** 1 Department of Psychology, University Bern, Bern, Switzerland; 2 Department of Neurology, Inselspital Bern, Bern, Switzerland; 3 Department of Neurology, Ludwig-Maximilians-Universität München, München, Germany; 4 Hertie Senior Professor for Clinical Neuroscience, Ludwig-Maximilians- Universität München, München, Germany; 5 German Center for Vertigo and Balance Disorders-IFBLMU (DSGZ), Ludwig-Maximilians-Universität München, München, Germany; 6 Munich Cluster for Systems Neurology (SyNergy), Munich, Germany

**Keywords:** Cognitive neuroscience, Human behaviour

## Abstract

A distorted model of a familiar multi-level building with a systematic overestimation of the height was demonstrated earlier in psychophysical and real world navigational tasks. In the current study we further investigated this phenomenon with a tablet-based application. Participants were asked to adjust height and width of the presented buildings to best match their memory of the dimensional ratio. The estimation errors between adjusted and true height-width ratios were analyzed. Additionally, familiarity with respect to in- and outside of the building as well as demographic data were acquired. A total of 142 subjects aged 21 to 90 years from the cities of Bern and Munich were tested. Major results were: (1) a median overestimation of the height of the multi-level buildings of 11%; (2) estimation errors were significantly less if the particular building was unknown to participants; (3) in contrast, the height of tower-like buildings was underestimated; (4) the height of long, flat shaped buildings was overestimated. (5) Further features, such as the architectonical complexity were critical. Overall, our internal models of large multi-level buildings are distorted due to multiple factors including geometric features and memory effects demonstrating that such individual models are not rigid but plastic with consequences for spatial orientation and navigation.

## Introduction

Recent, psychophysical and navigational real world experiments showed that the representation of 3-D buildings and the navigation performance within them is anisotropic in humans^1,2^. For example it was demonstrated using a behavioral pointing task that the internal representation of a large multilevel building was highly distorted^1^. In the pointing study, only long-time employees who worked in the university clinic on a daily basis for multiple years were asked to point at various landmarks, e.g. lecture halls or certain wards. Based on the pointing directions a 3 dimensional model of the internal representation of the building was calculated to construct a mathematical model. The results revealed that the mental model of the building was represented 215% taller/higher and 51% shorter in width than its actual physical dimensions.

A subsequent study compared the performance in vertical and horizontal real navigation in the same multilevel building. The subjects were instructed to find items, previously visited during a guided walk, in a horizontal and vertical (stairways) condition. As expected for a ground based species the navigation performance was better for the horizontal plane^2^. A similar anisotropy has been reported for the representations of passively traveled distances. In the study, the participants had to estimate the perceived travelled distance relative to a reference stimulus. It was found that the estimation-errors depended on the movement direction and the authors further concluded that the anisotropy was more likely to be body rather than earth-centered^3^. Biases towards the horizontal plane have also been reported in other ground based animals such as canine^4^ and rat^5^. No biases were found in swimming and flying species which move in a 3-D environment using path integration, continuously integrating direction and distance to envisaged goals^6,7^.

Spatial distortions of internal models have also been addressed in other fields of research. For example a study on face recognition used an image rescaling procedure to access the internal model of familiar and unfamiliar faces^8^. Interestingly, no significant differences in the error magnitude (8–13%) were found between the two categories.

The goal of the current study was to investigate whether the reported effect of height overestimation of large multi-level buildings is a general phenomenon with relevance for orientation and navigation. This experiment was missing, since the mathematical model calculated from the pointing data within the building^1^ and the interpretation of an anisotropy is not necessarily a proof that we really use mental (distorted) models of the entire building for pointing on invisible targets or navigating in a multi-level environment. In the current experiment we asked the participants to actively compare the global shape of buildings on a screen with the memorized shape. More specifically, we investigated whether the spatial anisotropic representation of multilevel buildings depends on the grade of familiarization with the building and whether there are any age or gender effects. We conducted a tablet-based experiment testing healthy volunteers in two European cities.

## Methods

### Participants

A total of 142 healthy volunteers (81 female, 61 male) with a mean age of 33.7 years ranging from 21 to 90 years (SD = 13.3) from the cities of Bern (N = 74; 38 female, 36 male; 22–90 years; mean = 34.3 years; SD = 14.8 years) and Munich (N = 68; 43 female, 25 male; 21–68 years; mean = 33.1 years; SD = 11.0 years) participated in this study. 125 participants reported to be right-handed, 10 to be left-handed, and 7 stated to be ambidextrous. 59.9% of the participants used glasses or contact lenses during the experiment. Most of the tested persons (58.8%) had a university degree, 18 (12.7%) had a degree from a university of applied science, 22 (15.5%) graduated from high school, and 20 (14.1%) had a secondary school diploma. This study was carried out in accordance with the Helsinki Declaration and approved by the Ethics Committee of the Human Sciences Faculty of the University of Bern as well as the Institutional Review Board of the ethics committee of the Ludwig-Maximilians University Munich. All participants gave their informed consent before the experiment.

### Images

**Figure 1:**
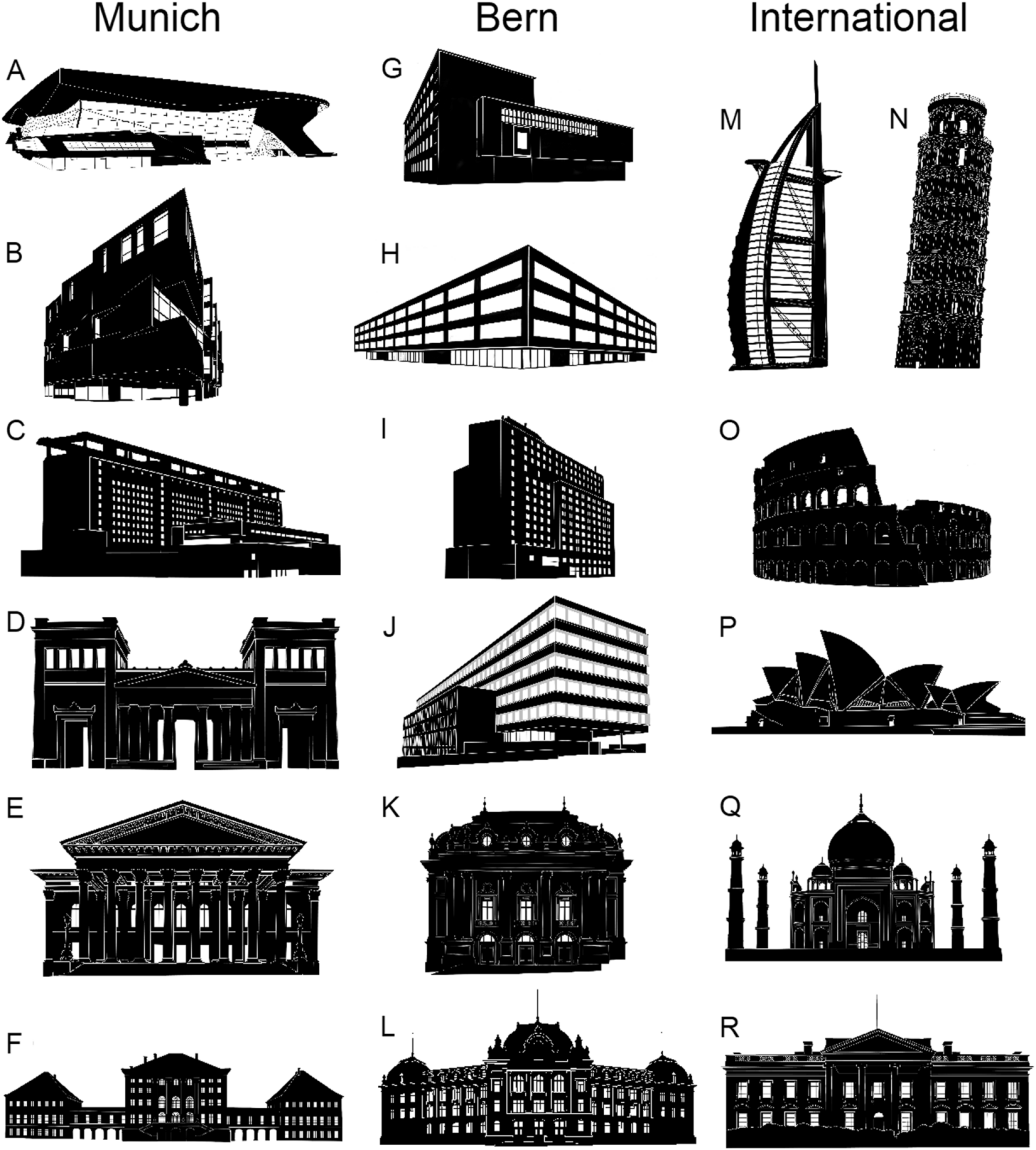
Buildings from the cities of Munich and Bern as well as international Buildings were used in this study. Buildings from Munich were (**A**) BMW-World, (**B**) Galeria-Building, (**C**) University Hospital, (**D**) Propylaea, (**E**) National Theatre, (**F**) Nymphenburg Castle. Buildings from Bern were (**G**) Natural History Museum, (**H**) von Roll Building, (**I**) IBIS Hotel, (**J**) Welle 7, (**K**) Bern Theatre, (**L**) University Building. The international buildings were (**M**) Burj al Arab, Dubai UAE, (**N**) Tower of Pisa, Pisa, Italy, (**O**) Colosseum, Rome, Italy (**P**) Sydney Opera House, Sydney, Australia (**Q**) Taj Mahal, Agra, India (**R**) White House, Washington DC, USA. All buildings were presented twice in random order.

In total 18 pictures from three categories were presented in this study (Fig. 1). The first category consisted of six famous international buildings, namely the Burj al Arab, the Colosseum, the Sydney Opera House, the Taj Mahal, the Tower of Pisa, and the White House. The pictures of the second category showed well-known buildings located in the city of Munich, Germany (BMW-World, University Hospital University Hospital, Galeria-Building, National Theater, Nymphenburg Palace, Propylaea). The third category consisted of well-known buildings located in Bern, Switzerland (Bern Theatre, IBIS Hotel, Natural History Museum, University Building, von Roll Building, Welle 7). The pictures of the buildings were processed using Photoshop (Version CS6). At first the buildings were separated from the background and the silhouettes were set to be black. In a second step the details of the building were recovered by manual processing. Particularly, windows, doors, pillars, ornaments, and other distinctive features were added in order to make the appearance of the building and perspective recognizable. We did intentionally not add certain features like captions, logos or circular objects because they provide a strong cue for distortions. However, we are not aware of any study systematically investigating if such features provide beneficial cues. Finally, the images were resized (cropped) to the minimum possible size around the edges of the outer contour of each building.

### Procedure

The experiment was implemented as a mobile application programmed in-house in the Java programming language and installed on a Lenovo Tab 10 Tablet. The experiment started by acquiring the following demographic data: age, gender, handedness, and question on the usage of visual aids during the experiment. The participants then performed twelve trials on six city specific buildings and six international buildings. Every trial started with the question whether the participant knows the displayed building (yes/no) followed by the question how familiar they are with the inside of the building (scale: 1–5). Then, the participants were asked to adjust the height/width ratio of the displayed building to fit best with their memory or imagination using two sliders for the x-axis (width) and y-axis (height) respectively. In other words, the participants were asked to generate a width/height ratio rather than to estimate absolute values for width and height. Each building was presented twice, once with the width/height ratio smaller than the true value (squeezed) and once stretched along the vertical axis with the initial width/height ratio exceeding the correct value. The magnitude of distortion and the order of starting condition (squeezed/stretched) were randomized for each trial. In the stretched condition the maximum height was between 300 and 600 pixels while the width between 150 and 300 pixel and vice versa in the squeezed condition. Similarly, the subjects could only adjust the buildings to a maximum of 600 pixels in each direction. This set of parameters was chosen because pilot runs showed that this results in initial distortions that are not too extreme. Particularly, it was ensured, that the initial image could be recognized as a building. The goal of presenting the images distorted in both ways was to rule out possible biases introduced by the initial appearance. Participants did not receive any feedback and had no time limit for the adjustment. The pictures were presented in a randomized order. Participants did not receive any feedback and had no time limit for the adjustment. The pictures were presented in randomized order.

## Results

**Figure 2:**
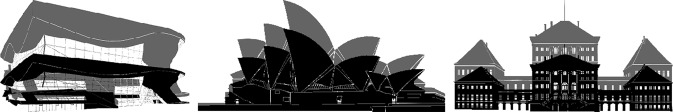
Visualization of the estimation errors for the BMW-World (left), Sydney Opera House (center), and Nymphenburg Castle (right). The black building represents the true ratio and the grey building shows the median adjustment by all participants. The three displayed buildings do not have a traditional floor concept or roof structure and are among the ones with the largest errors of all tested buildings.

**Figure 3:**
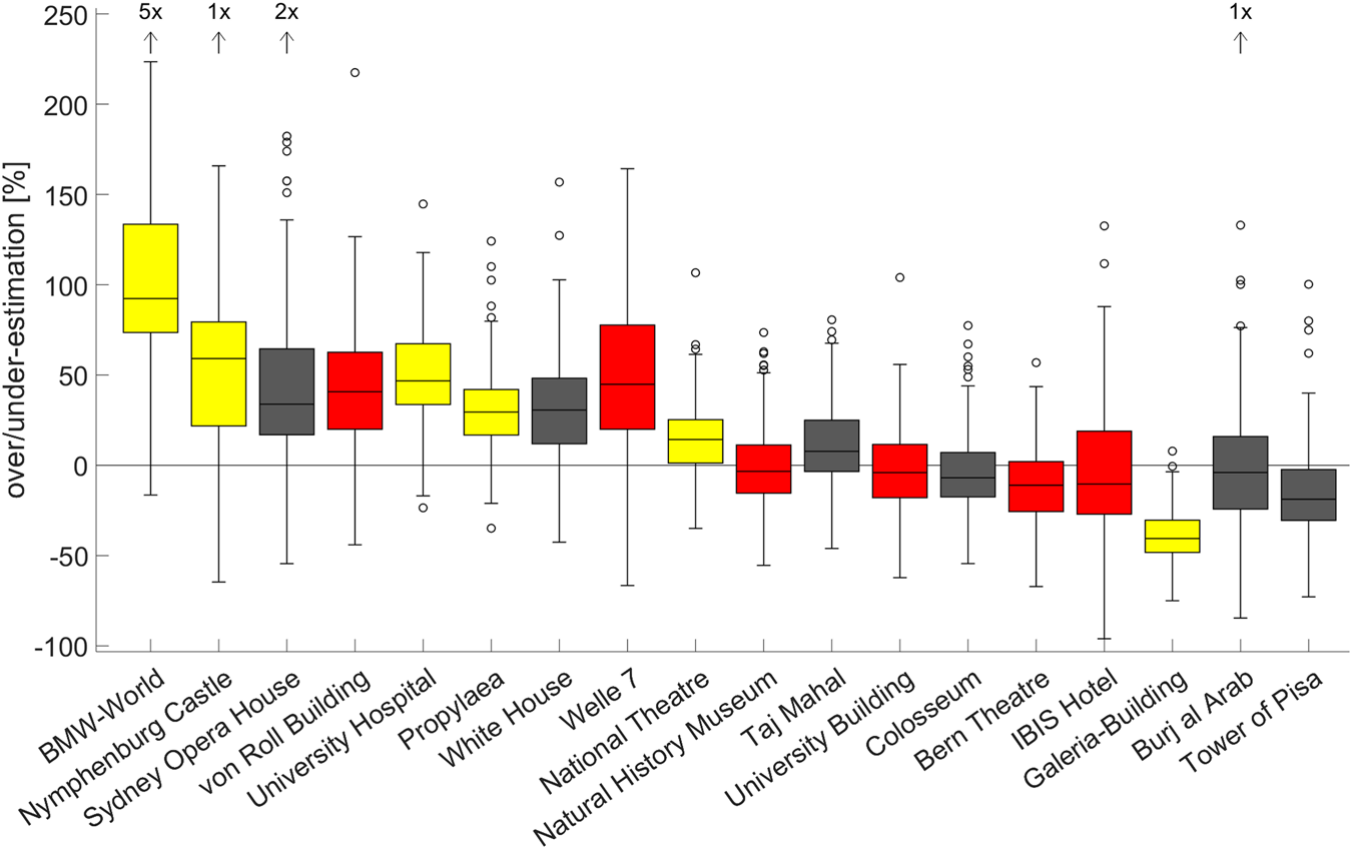
Estimation errors in percentage for every building from Munich (yellow), Bern (red), and international locations (grey). The buildings are ordered by their height/width-ratio with wide buildings on the left and the tall buildings on the right (Table 1). The boxes contain 50% of all measured data points, the black horizontal lines represent the median and the whiskers indicate the 95^th^ percentile. Outliers are represented as circles. The number of data points not visible due to the scaling are mentioned on top of the arrows.

The median across all buildings (Fig. 1) showed a median overestimation of the height by 11% (SD: 36%) ranging from −41% to 92% with no significant difference between female and male subjects (p = 0.065). The median values (height/width) for the different buildings were as follows and are visualized in Figs 2 and 3 and summarized in Table 1. If the estimation errors are calculated with respect to the short/long ratio, rather than the height-width ratio, the median error is 12% (SD: 47%). This increased median errors point towards a key role of the short/long dimension ratio during the estimation process. Comparing the estimation error relative to the initial ratios (squeezed or stretched) a significant (p < 0.001; z-val: 6.07; r = 0.104) was found using a Wilcoxon rank sum test. The median estimation errors was 6.8% from a squeezed and 15% from a stretched starting condition.

**Table 1:** Median estimation errors in percentage for every building of the study.

Building	median error (SD) [%]	image ratio (h/w)
Burj al Arab	−4.0 (40)	2.72
Taj Mahal	7.8 (23)	0.61
Sydney Opera House	33 (54)	0.34
Tower of Pisa	−19 (25)	3.05
Colosseum	6.9 (21)	0.70
White House	30 (30)	0.49
University Hospital	47 (32)	0.46
BMW-World	92 (108)	0.31
National Theatre	14 (25)	0.58
Galeria-Building	−41 (15)	1.22
Propylaea	39 (27)	0.49
Nymphenburg Castle	59 (57)	0.32
IBIS Hotel	−10 (39)	0.96
Natural History Museum	−3.4 (27)	0.59
Bern Theatre	−11 (20)	0.84
University Building	−4 (24)	0.67
von Roll Building	41 (34)	0.41
Welle 7	45 (36)	0.49

Additionally, the height-width (h/w) ratio of the original image is provided.

Comparing the results for images from the three different locations (Bern, Munich, International) a significant difference was found using a Kruskal-Wallis test (p < 0.001; Chi-sq = 73.3, Freeman’s theta = 0.17). The post-hoc Wilcoxon rank sum tests showed significantly larger errors for buildings in Munich compared to international (p < 0.001; z-val: 8.44, r = 0.24) or buildings from Bern (p < 0.001; z-val: 7.00, r = 0.23). No difference was found for buildings from Bern and international locations (p = 0.74; z-val: 0.33). Comparing the performance between the two cohorts for the international buildings no significant difference was observed (p = 0.42; z-val: 0.81).

Testing the median errors for buildings reported to be unfamiliar (N = 846) to those reported to be known (N = 1708) by the subjects (i.e., either seen or had entered the building in reality) a significantly smaller error (p < 0.001; z-val: 3.76, Freemann’s theta = 0.14) was observed for unknown buildings across all subjects. The pattern was also detectable separately for both cohorts but did only reach the significance level in Bern (p = 0.005; z-val: −2.83) but not Munich (p = 0.114; z-val: 1.58).

For the buildings reported to be known by the subjects possible differences between the barely known (rated 1) up to the well-known (rated 5). In total most buildings (N = 1069) were rated familiarity 1 by the participants, while the other ratings were selected rarely (rating 2: 204; rating 3: 190; rating 4: 125; rating 5: 86. A Kruskal-Wallis test indicated significant (p = 0.009; Chi-sq: 15.4, Freeman’s theta = 0.09) differences between the familiarity ratings. The post-hoc Wilcoxon rank sum tests were significant between the ratings 1 vs. 2 (p < 0.001; z-val: 3.31, r = 0.09) and 1 vs. 4 (p = 0.016; z-val: 2.40, r = 0.07), showing significantly reduced estimation errors for the least known buildings.

**Figure 4:**
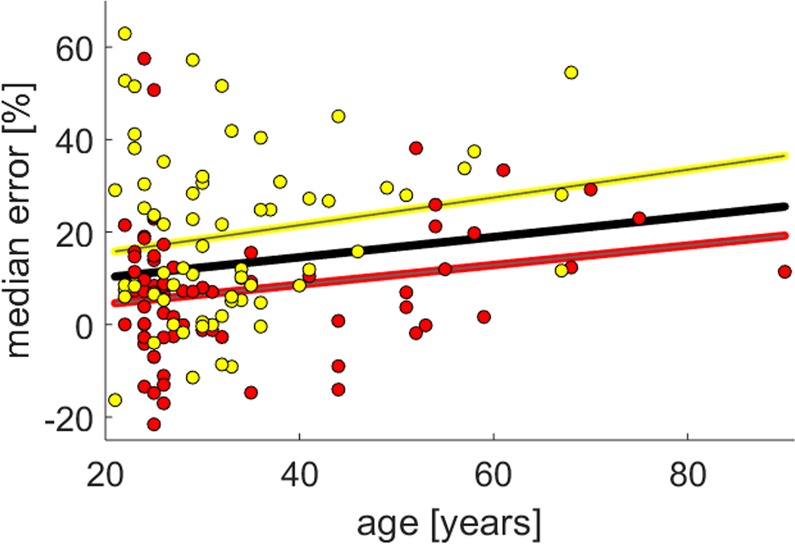
The graph shows the correlation between the age and the median error. For all subjects the correlation of r = 0.167 was significant (p = 0.047). For the two subgroups from Bern (red) and Munich (yellow) the correlation between age and median estimation error were roughly the same (r_Bern_ = 0.219; r_Munich_ = 0.183) but they did not reach the significance level of 0.05 (p_Bern_ = 0.060; p_Munich_ = 0.136).

Additionally, a relatively weak but significant correlation (p = 0.047; r = 0.167) between the median estimation error and the age of the subject could be found (Fig. 4). The correlation coefficients for the subgroup from Bern r = 0.219 and Munich r = 0.183 did not reach p values smaller than 0.05 (p_Bern_ = 0.06; p_Munich_ = 0.14). For the relationship between age and familiar buildings we found a correlation of r = 0.299 (p = 0.002) but the correlation between age and unknown buildings was not significant (r = 0.065; p = 0.509 ).

## Discussion

In this study, it was tested whether the finding of a systematic overestimation of a multilevel hospital building generalizes to other familiar and unfamiliar multilevel buildings. This was the case with a median overestimation of the height of 11% (−41% up to 92%) for local (Bern, Munich) and international buildings. However, there were variations depending on architectonical features, age, and the question whether the participants had seen the real building or photos/drawings/movies. The height of tower-like buildings was underestimated, whereas that of long, flat shaped buildings was overestimated. The longest dimension of a multilevel building was underestimated independent of its earth- vertical or -horizontal orientation. Estimation errors were significantly less if the particular building was unfamiliar to participants. The overestimation of height of familiar buildings in which the subjects spend years of their professional life was most pronounced. This is evident from the comparison of the degree of misperception of familiar city specific versus less familiar international buildings. The architectonical complexity or the perspective of the presentation were also critical for example the overestimation of the height of the Sydney opera or the BMW-World (Fig. 2). Gender had no systematic influence on the performance. Overall, our internal models of large multi-level buildings are distorted due to multiple factors including geometric features and memory effects.

A significant overestimation along the vertical axis and underestimation along the horizontal axis is in agreement with the known “vertical-horizontal illusion” and was consistently reproduced in psychological experiments mainly by means of simple geometric figures like drawings^9^. There were also experiments showing that the same illusion was evident in visually-rich environments with real world objects up to 18.6 m (61 ft.)^10^. The overall mean error across all tested objects was about +8%. Yang and co-workers^11^ already described two important features of this illusion which are also relevant for our study: first, that overestimation was greater when viewing real objects than objects in pictures and, second, that the effect was larger for big as compared to small objects.

We found that the heights of towers or tower-like buildings (Tower of Pisa, Burj al Arab) with an original picture height-width ratio larger than 2.7 are on average underestimated by 11.4% (Table 1). Interestingly, the height of long buildings with small height-width ratios like the university hospital in Munich, Welle 7, and the von Roll building were typically overestimated. The common feature among these finding seems to be a systematic underestimation of the longest dimension (height or width) and it is therefore tempting to conclude that humans are biased towards equalizing height and width (Fig. 3).

Additionally, to the long-short dimension ratio further factors seem to play a key-role. Comparing our results of the sub analyses for the estimations of the different buildings a complex interrelation emerged and a single median estimation error is not sufficient for describing the complexity of the obtained results. An important factor impacting the estimation error is the familiarity with the building. While subjects unfamiliar with the building were on average able to estimate the height width ratio of the buildings with a small median error, the misperception was greater for known buildings. There is a fundamental difference of the two tasks, the comparison of two-dimensional pictures (which is the case with unfamiliar buildings) and the comparison of the internal model of a familiar building which has been experienced by real exploration and navigation. It appears that the internal model changes its dimensions by long-term personal interaction of the individual with the building. Future neuroimaging studies will reveal whether the bias for familiar buildings involves different neuronal networks including hippocampal and parietal lobe structures for human navigation and orientation when compared to buildings that have only been seen but never entered. A similar surprising result was observed in a face recognition study^8^, where participants adjusted the size of familiar and unfamiliar faces and logos. While for the familiar logos the errors were smaller, no advantage was found for the familiar faces in two out of three experiments and the errors were significantly larger for familiar faces in the third experiment. It is also worth mentioning that the authors reported no adjustment effects relative to the starting conditions while such an effect was observed for the buildings used in this study.

Another factor influencing the ratio estimation was the presence of a visible regular floor concept. Buildings with complex, less organized structures were significantly overestimated in height. In our stimuli set the BMW-World, the Propylaea, the Nymphenburg Castle, and the Sydney Opera are examples of buildings with such complex overall or roof structures and a non-standard floor concept. These buildings are among the buildings with the largest estimation errors (33–92%).

A further factor depends on the perspective of the building. This was best demonstrated by the Galeria-Building in Munich, a large department store. The picture differs from the others in that it was taken on ground level from a relatively short distance with the camera pointing slightly upwards. We speculate that this unique perspective explains the strong underestimation of the building’s height. The impact of perspective on the perception of 2D images is a current and interesting field of research^12–14^. An extensive discussion regarding the relationship between physical, perspective and the retinal image can be found elsewhere^15^.

All factors mentioned above need an independent confirmation as the number of buildings in this study is relatively small and a systematic categorization based on architectural features were not consequently considered in the design of the study. However, the three factors (ratio, complexity, and perspective) are helpful to understand the significantly smaller errors in Bern compared to the Munich cohort for the national buildings and the absence of a difference for the international buildings. The differences between the city specific picture sets can most objectively been demonstrated by comparing the average ratio between the long and short dimension (Munich 2.26; Bern 1.65) which are considerably larger for the buildings from Munich.

In the current study a significant increase of the estimation error with age was found. This is in line with the action-specific perception model which states that people perceive their environment in terms of their ability to act in it^16^. Given the reduced mobility of elder people a pronounced overestimation of buildings might reflect an altered action-cost function^17^. Such altered action-cost functions were already observed in experiments on the distance-on-hill effect^18^, or when the effort of movements was manipulated by additional weights^19^. The phenomenon of a systematic overestimation of height in less mobile participants has also recently been reported in a study that compared height of a wall estimates of a group of traceurs and novices^20^.

The finding of a systematic misjudgment of the height of multi-level buildings up to 92% can be of some importance in emergency situations. For example in the case of a house fire the wrong estimation in combination with the decreased vertical navigation skills can lead to wrong decisions when visual cues are impoverished, e.g., searching for the exit at a wrong floor due to an internal misrepresentation of the building.

In conclusion, the data support the view that our internal model of large buildings which we know from direct personal interactions, from pictures or other visual media are distorted. The models are not rigid but plastic depending on various features. What they have in common is misperception in form of an underestimation of the longest dimension (height of towers) and an overestimation of the shortest dimension (most multilevel buildings). This anisotropy of misperception has analogues to spatial orientation and navigation.

## Data Availability

The datasets generated during and/or analyzed during the current study are available from the corresponding author on reasonable request.
